# High-Density Single Nucleotide Polymorphisms Genetic Map Construction and Quantitative Trait Locus Mapping of Color-Related Traits of Purple Sweet Potato [*Ipomoea batatas* (L.) Lam.]

**DOI:** 10.3389/fpls.2021.797041

**Published:** 2022-01-06

**Authors:** Hui Yan, Meng Ma, Muhammad Qadir Ahmad, Mohamed Hamed Arisha, Wei Tang, Chen Li, Yungang Zhang, Meng Kou, Xin Wang, Runfei Gao, Weihan Song, Zongyun Li, Qiang Li

**Affiliations:** ^1^Institute of Integrative Plant Biology, Jiangsu Key Laboratory of Phylogenomics and Comparative Genomics, School of Life Sciences, Jiangsu Normal University, Xuzhou, China; ^2^Xuzhou Institute of Agricultural Sciences in Jiangsu Xuhuai District, Key Laboratory of Biology and Genetic Improvement of Sweetpotato, Ministry of Agriculture, Sweetpotato Research Institute, Chinese Academy of Agricultural Sciences (CAAS), Xuzhou, China; ^3^Department of Plant Breeding and Genetics, Bahauddin Zakariya University, Multan, Pakistan; ^4^Department of Horticulture, Faculty of Agriculture, Zagazig University, Zagazig, Egypt

**Keywords:** sweet potato, genetic linkage map, flesh color, anthocyanin content, QTL, SNP

## Abstract

Flesh color (FC), skin color (SC), and anthocyanin content (AC) are three important traits being used for commodity evaluation in purple-fleshed sweet potato. However, to date, only a few reports are available on the inheritance of these traits. In this study, we used a biparental mapping population of 274 F1 progeny generated from a cross between a dark purple-fleshed (Xuzishu8) and white-fleshed (Meiguohong) sweet potato variety for genetic analyses. Correlation analysis showed a significant positive correlation among AC, SC, and FC. Medium-to-high heritability was observed for these traits. We detected single nucleotide polymorphisms (SNPs) by specific length amplified fragment sequencing (SLAF-seq) with the average sequencing depth of 51.72 and 25.76 for parents and progeny, respectively. Then we constructed an integrated genetic map consisting of 15 linkage groups (LGS) of sweet potato spanning on 2,233.66 cm with an average map distance of 0.71 cm between adjacent markers. Based on the linkage map, ten major quantitative trait loci (QTLs) associated to FC, SC, and AC were identified on LG12 between 0 and 64.97 cm distance, such as one QTL for SC and FC, respectively, which explained 36.3 and 45.9% of phenotypic variation; eight QTLs for AC, which explained 10.5–28.5% of the variation. These major QTLs were highly consistent and co-localized on LG12. Positive correlation, high heritability, and co-localization of QTLs on the same LG group confirm the significance of this study to establish a marker-assisted breeding program for sweet potato improvement.

## Introduction

Sweet potato [*Ipomoea batatas* (L.) Lam., *I. batatas* (2n = 6x = 90)] is an important root crop worldwide owing to its rich nutritional contents, high yield, and wide range of adaptability (Ma et al., 2020). However, genetic studies in sweet potatoes are significantly lagging behind other major diploid crops due to their high degree of genetic heterozygosity and self-incompatibility.

A high-quality and high-density genetic linkage map represents an important tool for quantitative trait loci (QTLs) mapping, especially for complex quantitative traits. Additionally, the availability of numerous stable and reliable molecular markers is the key and foundation for constructing a high-quality genetic map. In the past three decades, various types of molecular markers have been developed and utilized by researchers for crop improvement purposes, such as random amplified polymorphic DNA (RAPD) (Ukoskit et al., 1997), amplified fragment length polymorphism (AFLP) (Kriegner et al., 2003; Cervantes-Flores et al., 2008; Zhao et al., 2013; Ma et al., 2020), simple sequence repeats (SSR) (Zhao et al., 2013; Kim et al., 2017; Ma et al., 2020), and single-nucleotide polymorphism (SNP) (Zhang Z. et al., 2015; Shirasawa et al., 2017; Mollinari et al., 2020). However, the utilization of traditional markers, such as AFLPs and RAPDs, is restricted due to their limited quantity, repeatability, and stability (Yagi, 2018).

Recently, the identification of SNP markers based on next-generation sequencing (NGS) technology has been considered ideal molecular marker types for high-density genetic map construction in crop plants. In sweet potato, Shirasawa et al. (2017) constructed the first SNP linkage map using the S1 population of “Xushu18” which included 96 linkage groups (LGs) with an average distance of 1.18 cm (Shirasawa et al., 2017). Furthermore, Mollinari et al. (2020) also constructed a high-density SNP genetic map in sweet potato for beta-carotene and dry matter contents. The genetic map included 15 LGs with a total and average genetic map distance of 2,708.3 and 0.09 cm, respectively.

The specific length amplified fragment sequencing (SLAF-seq) is a double pseudo-test cross strategy of high throughput genome sequencing (Guo et al., 2015). SLAF-seq has been successfully applied for the development of high-throughput SNP markers for polyploid species, such as walnut, upland cotton, and chrysanthemum (Zhang J. et al., 2015; Zhu et al., 2015; Song et al., 2020). Using the SLAF-seq technology, we developed SNP markers and constructed a high-density and high-quality genetic linkage map for sweet potato, which represents an important tool for QTL mapping, especially for complex quantitative traits such as anthocyanin content (AC).

Quantitative trait locus analysis based on genetic linkage maps is a valuable and effective method for mining tightly linked molecular markers and determining the position of genes controlling the traits of interest. Flesh color (FC), skin color (SC), and AC content are three important color-related traits of purple sweet potato. Previously, some studies have reported the presence of QTL significantly associated with FC in sweet potato on LG3 and LG12 (Zhang et al., 2020). However, the studies on the genetic inheritance of SC and AC are limited. In addition, systematic studies on the correlation among these traits are also limited. Therefore, in this study, we have determined the nature of association among these traits over 2 years of phenotyping. Furthermore, QTL analysis was performed to illuminate the genetic mechanisms underlying AC, FC, and SC.

## Materials and Methods

### Mapping Population Construction and DNA Extraction

The mapping population comprising of 274 F_1_ progeny was derived from crossing a dark purple-fleshed sweet potato cultivar “Xuzishu8” (female parent) and white-fleshed variety “Meiguohong” (male parent). These two cultivars were selected due to their agronomic importance and contrasting traits, such as FC and AC. Crossing by hand pollination was performed to generate F_1_ seeds during June–September 2016. The F_1_ hybrid seeds were directly sown in the greenhouse in May 2017. Young plants were transferred to the experimental field at the Xuzhou Institute of Agricultural Sciences in Jiangsu Xuhuai District (117°45′34″E, 34°44′26″N), Jiangsu, China in June 2017.

A total of 274 individuals and two parental cultivars were used for genetic analysis. The total DNA of the F_1_ lines and both the parents were extracted from fresh healthy young leaves using the plant Genomic DNA Extraction Kit (Tiangen Biotech Co., Beijing, China). The quantity and quality of genomic DNA were analyzed using NanoDrop 2000 spectrophotometer (NanoDrop, Wilmington, DE, United States) and 1% agarose gel electrophoresis where ƛDNA was used as a check.

### Field Experiments and Phenotyping of Traits

F_1_ hybrids along with their parents were phenotyped during 2019 and 2020 in the experimental research station of the Xuzhou Institute of Agricultural Sciences at Yuanqu (YQ) and Zhaotuan (ZT). For precise phenotyping, multi-year and multi-locational trials were conducted. SC was divided into yellow, red, and purple colors by visual inspection. And FC was divided into white, yellow, and purple by visual inspection. AC contents were determined by spectrophotometer using the modified method as suggested by Tang et al. (2018).

The experiment was conducted in a randomized complete block design (RCBD). Each progeny was replicated thrice, and each replication had 10 plants per entry. The ridge width, length, and distance between ridges were 0.85, 4.5, and 0.9 m, respectively. Experimental material was planted on June 28 and harvested on October 28, with a growth period of 122 d.

ANOVA, correlation, and heritability in a broad sense (H^2^) were determined using ICIM 4.2 mapping software (Meng et al., 2015). Heritability in a broad sense on the basis of the mean across replications and environments (or heritability per mean) was estimated by the following formula (Zhao et al., 2013; Meng et al., 2015):


$$H^{2}=\frac{V_{G}}{V_{G}+\frac{1}{e}\,V_{\mathrm{GE}}+\frac{1}{r\,e}\,V\,\varepsilon }$$

### Library Construction, Illumina Sequencing, and Data Filtering

Genotyping of two parents was performed using whole genome sequencing (WGS). The average sequencing depth of each parent was 50×, and the clean data were not less than 40 G. Genomic DNA was randomly broken by ultrasonic fragmentation into 200–500 bp fragments. These DNA fragments were then ligated for end repair using 3‘A and sequence connectors followed by purification and amplification by PCR to complete the construction of the sequence library.

An improved SLAF-seq strategy was adopted to genotype the 274 F_1_ progenies as described previously by Sun et al. (2013). According to the size of the sweet potato genome (Yang et al., 2017) and GC content, the *Ipomoea trifida* (*I. trifida*) was finally selected as the reference genome to perform an enzyme digestion prediction (Hirakawa et al., 2015; Wu et al., 2018). Enzyme Rsal (New England Biolabs, NEB, Ipswich, MA United States) was used to digest the genomic DNA. The digested fragment was then subjected to 3′ end plus A (nucleotide) treatment, and a dual index (PAGE-purified, Life Technologies, Waltham, MA, United States) sequencing linker was ligated to the A-tailed fragment, followed by PCR amplification. Fragments ranging from 264 to 364 bp in size were excised and purified using a QIA quick gel extraction kit (Qiagen, Hilden, Germany). The DNA obtained by gel recovery was used for performing sequencing on an Illumina HiSeq 2500 system (Illumina, Inc., San Diego, CA, United States) according to the recommendations of the manufacturer. To monitor the accuracy of library construction and the sequencing test, the same protocol was followed for the genomic DNA of *Oryza sativa* L. japonica^1^ as a control trial.

### Sequence Data Grouping and Genotyping

Specific length amplified fragment marker identification and genotyping were performed using procedures described by Sun et al. (2013). According to the barcode sequences, raw reads were assigned to 274 individuals. High-quality reads with quality scores (QC) > 30 were identified for quality control. After the barcodes were trimmed from each high-quality read, these reads were mapped onto the *I. trifida* genome sequence by using SOAP software (Li et al., 2008; Li and Durbin, 2009). Sequences mapped to the same position with over 95% identity were defined as one SLAF locus (Zhang et al., 2015). SNP loci of each SLAF locus were then detected between parents, and the SLAFs with more than 3 SNPs were filtered out. Then the alleles of each SLAF locus were defined. All polymorphism SLAF loci were genotyped with consistency in the parental and offspring SNP loci. The marker code of the polymorphic SLAFs was analyzed according to the cross-pollinator (CP) population type, which consists of five segregation types (ab × cd, ef × eg, hk × hk, lm × ll, and nn × np) (Zhang et al., 2015).

Genotype scoring was performed using a Bayesian approach to further ensure the genotyping quality (Sun et al., 2013). High-quality SLAF markers were filtered out using the following criteria. SNPs with average sequence depths > 2-fold in each progeny and > 10-fold in the parents and with more than 75% missing data were filtered out. Then, the chi-square test was performed to examine the segregation distortion. Markers with significant segregation distortion (*P* < 0.05) were initially excluded from the map construction and were then added later as accessory markers.

### Genetic Linkage Map Construction

The SNP markers loci were arranged into LGs based on their locations on *the I. trifida* genome. The Modified logarithm of odd (MLOD) scores between markers was calculated to further confirm the robustness of markers for each LGs. Markers with MLOD scores < 3 were filtered out prior to ordering. To ensure efficient construction of the high-density and high-quality map, HighMap (Liu et al., 2014) strategy was adopted to order the SNP markers and genotyping errors correction within LGs. The consensus map was established by integrating the parental maps through the anchor markers (markers that were heterozygous in both parents). For anchor markers, map distance was calculated as the average distance between both parental distances. The remaining markers segregating in only one of the parents were placed on the consensus map by interpolation or extrapolation according to the relative position between the flanking anchor markers on the relevant parental map. Map distances were estimated using the Kosambi mapping function (Kosambi et al., 1943).

### Genetic Map Evaluation

The quality of the genetic map was evaluated by individual integrity, haplotype maps, heatmap, and Spearman rank correlation coefficient. Haplotype map was used to detect double crossover populations and suggest genotyping errors. Heatmap was used to evaluate the relationship of recombination between markers from each LG. Using the *I. trifida* reference (Wu et al., 2018), we detected collinearity blocks within each LG.

### Quantitative Trait Loci Mapping and Genome-Wide Association Study

Using the 15 LGs of the constructed integrated map, QTL analysis of three color-related traits was performed by the R/qtl package (Broman et al., 2003; Li et al., 2007). The LOD threshold for declaring a QTL significant was calculated by using a mapping step of 1.0 cm and 1,000 permutation tests at a significance level of *P* < 0.05. Then this LOD threshold value was used to detect QTLs significantly associated with color-related traits by composite interval mapping (CIM) model, and QTLs in every 1 cm interval in each LG were scanned and their contribution rate was calculated. For this purpose, anchored reference genomes of diploid relatives of sweet potato, *I. trifida* was used (Wu et al., 2018). QTLs with LOD values ≥ 2.5 were considered significant.

For higher reliability of QTL results, a genome-wide association study (GWAS) was performed for AC contents using genome-wide efficient mixed-model analysis for association study (GEMMA) (Zhou and Stephens, 2012). Pedigree relationship among individuals of the bi-parental population was dealt with using GEMMA software which accounts for genetic relatedness/pedigree among individuals as a K matrix. The overall association study was conducted following formulae given below:

y = Wα + xβ + μ+ e. Where μ is used as a random effect, W is as a covariate used as a fixed effect, X is the genotype, and Y is the phenotype. Manhattan plot and quantile-quantile (Q-Q) plot were constructed using the qqman R package. The Bonferroni correction for multiple testing was performed using an R script (Turner, 2014), and SNPs with a significantly high relationship to AC content were identified.

## Results

### Phenotypic Performance of Tuberous-Related Traits in the F_1_ Population

**FIGURE 1:**
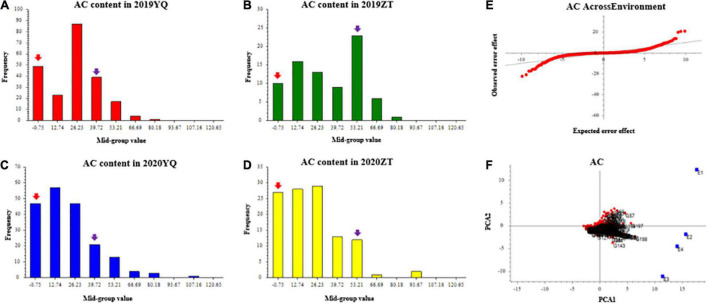
The frequency distribution of anthocyanin content of mapping population in YQ and ZT experimental plantations in 2019 and 2020. Panels **(A–D)** were the AC distributions in different plots. The x-axis indicates the AC content; the y-axis indicates frequency. The red and purple arrows indicate the mean value of “Meiguohong” and “Xuzishu8,” respectively. **(E)** Quantile-quantile plot of AC across environment; **(F)** GGE bi-plot of AC. The AC content in four experiments (E1: 2019YQ, E2: 2019ZT, E3: 2020YQ, E4: 2020ZT) was the average of three blocks. AC, anthocyanin content.

Three color-related traits, such as AC, SC, and FC, were measured for two consecutive years 2019 and 2020. AC varied continuously and showed transgressive segregation in the F1 population, which indicates that AC content has quantitative inheritance (Figures 1A–D). Q-Q plot of AC across environments showed AC with extreme values which confirms the presence of transgressive segregants in the population (Figure 1E). GGE bi-plot was used to analyze the genotype by environment interaction (GE). GGE method, i.e., genotypic effects + GE. The results showed that the AC content was predominantly conditioned by the genotype effect (Figure 1F).

**FIGURE 2:**
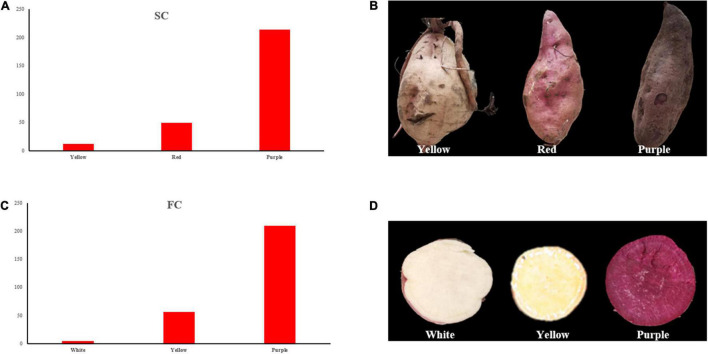
Frequency distribution of SC and FC. **(A)** SC frequency distribution of the individuals; **(B)** three skin color types of the individuals; **(C)** FC frequency distribution of the individuals; **(D)** three flesh color types of the individuals. SC, skin color; FC, Flesh color.

For SC, three different colors were observed in the offsprings, i.e., yellow, red, and purple (Figures 2A,B), and the segregation ratio of non-purple skin plants to purple skin plants has remained 3:1, which showed involvement of a dominant gene.

Flesh color showed white, yellow, and purple color individuals (Figures 2C,D), and the segregation ratio of non-purple plants to purple plants has also remained 3:1. The observed results of SC and FC were consistent in different experimental locations between 2019 and 2020.

Correlation analysis of AC, SC, and FC showed that AC contents were highly positively correlated with FC (*r* = 0.649) and SC (*r* = 0.585), and FC was highly positively correlated with SC (*r* = 0.874). ANOVA analysis of three traits in combined environments was supplied in Supplementary Table 1. A high value of heritability was observed for SC and FC. Anthocyanin content also showed a high value of the broad sense heritability (0.76) but less than SC and FC, which showed that AC content is environment dependent as compared to FC and SC (Table 1).

**TABLE 1:** Correlation and heritability analysis of color-related traits.

	Trait	AC	SC	FC	H^2^ by mean
YQ × ZT	AC	1.000	0.585***	0.649***	0.760
YQ × ZT	SC		1.000	0.874***	1
YQ × ZT	FC			1.000	1

***indicates significance at P ≤ 0.001.

*FC, flesh color; SC, skin color, and AC, anthocyanin content.*

### Analysis of SLAF-seq Data and SLAF Markers

**FIGURE 3:**
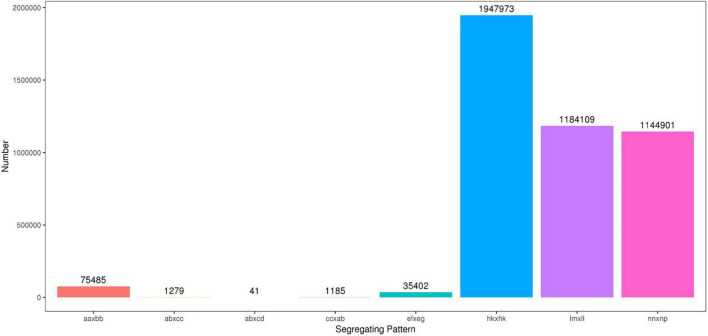
Number of polymorphic SNPs for eight segregation patterns. The x-axis indicates eight segregation patterns of polymorphic SNPs markers; the y-axis indicates the number of markers. SNPs, single nucleotide polymorphisms.

The high-throughput DNA sequencing generated a total of 1,442,547,436 pair-end reads with a Q30 value of 94.79% and GC content 41.24% indicating that good quality source data were generated (Table 2). Among these reads, 9,004,153 SNPs were detected, out of which, 4,390,375 were successfully genotyped. A total of 4,314,890 SNPs were used for genetic map construction, with an effective polymorphism rate of 47.92% of the total generated markers. The genotyped SNPs were classified into eight segregation types (Figure 3).

**TABLE 2:** Summary of the sequencing data for the two parents and F1 progeny.

Sample ID	Total reads	Number of SNPs	Q30 percentage (%)	GC percentage (%)	Average depth
Female “Xuzishu8”	138,822,581	4,468,412	94.53	37.33	51.71 X
Male “Meiguohong”	139,803,018	4,506,276	94.68	37.64	51.73 X
Offspring	4,247,889	1,115,793.99	94.79	41.27	25.76 X
Control	781,786	/	94.32	44.92	/
Total	1,442,547,436	9,004,153	94.79	41.24	/

Low-quality SNPs with a depth less than 10X in parents and segregation distortion (*P* < 0.01) were filtered out to ensure a set of high-quality SNPs. In this study, 4,151 high-quality SNP markers were selected for the construction of sweet potato genetic maps, such as the ef × eg (1), hk × hk (126), lm × ll (1984), and nn × np (2040) types. The average sequencing depth for both parents “Xuzishu8” and “Meiguohong” remained 51.71X and, 51.73X, respectively. Whereas in offspring average sequence depth has remained 25.76X for each F1 progeny.

### High-Density Genetic Map Construction and Its Basic Characteristics

Out of 4,151 SNPs, 3,923 high-quality SNPs on the basis of MLOD values between the two high-quality SNPs were successfully integrated into 15 LGs of sweet potato (Supplementary Table 2).

**FIGURE 4:**
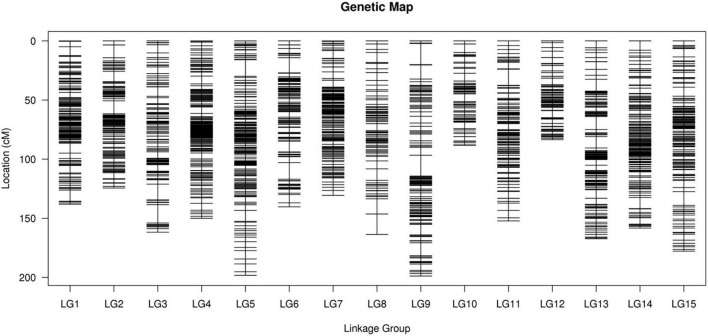
The integrated high-density genetic map of sweet potato based on 3,178 SNPs. The x-axis and y-axis represent linkage groups and location, respectively. SNPs, single nucleotide polymorphisms.

After performing linkage analysis, the final genetic map containing 3,178 markers was constructed by the integration of the parent’s genetic maps on the basis of the collinear sites in every LG (Table 3 and Figure 4).

**TABLE 3:** Summary of the 15 linkage groups of integrated sweet potato map.

Linkage group	MarkerNum	Total Distance	Average Distance	Gaps ≤ 5	Max Gap
LG01	249	138.17	0.56	98.39%	9.42
LG02	204	124.44	0.61	98.52%	10.92
LG03	203	161.79	0.80	96.04%	15.48
LG04	237	150.03	0.64	98.73%	6.57
LG05	236	198.24	0.84	98.3%	13.95
LG06	239	140.34	0.59	98.32%	14.05
LG07	211	130.63	0.62	98.57%	6.53
LG08	164	163.68	1.00	95.71%	17.31
LG09	239	198.85	0.84	96.22%	17.78
LG10	144	88.30	0.62	97.2%	7.20
LG11	177	152.22	0.86	96.59%	13.54
LG12	214	83.53	0.39	98.12%	5.94
LG13	231	167.34	0.73	96.96%	10.97
LG14	237	158.28	0.67	98.31%	9.35
LG15	193	177.82	0.93	97.92%	11.55
Total	3,178	2, 233.66	0.70	97.59%	17.78

The final map was 2,233.66 cm in length with an average distance of 0.70 cm. The genetic map of the female parent “Xuzishu8” consisted of 1,586 markers and was 2,051.33 cm in length with an average distance of 1.31 cm (Supplementary Table 3). A total of 1,700 markers were used for the construction of the genetic map of the male parent “Meiguohong,” with a total genetic map distance of 2,024.18 cm and an average distance of 1.20 cm (Supplementary Table 4). The average depth of the markers was 51.71X in the female parent, 51.73X in the male parent, and 25.76X in the offspring.

The number of SNP markers in the integrated map ranged from 144 (LG10, average distance of 0.62 cm) to 249 (LG01, average distance of 0.56 cm). The shortest LG was LG12, which contained 214 markers and exhibited a genetic map distance of 83.53 cm and an average inter marker distance of 0.39 cm. The longest LG was LG09, which contained 239 markers and exhibited a genetic map distance of 198.85 cm and an average inter marker distance of 0.84 cm. The largest and smallest genetic gap was also found in LG09 and LG12, which were 17.78 and 5.94 cm in length, separately. LG01 was the highest density group, which contained 249 markers, and the average marker density was 0.56 cm. LG10 was the lowest density group, which contained 144 markers, and the average marker density was 0.62 cm (Table 3).

According to a chi-square test, out of 3,178 markers, 96 markers on the map showed distorted segregations (*P* < 0.01) even though the extremely significant SNPs (*P* < 0.01) were excluded, and the total segregation distortion ratio was 3.02%. The greatest number of segregation distortion markers was occurred in LG12, with a segregation distortion ratio of 23.83%. There was no distorted marker in the other 10 linkage groups (Table 4).

**TABLE 4:** Distribution of distorted segregation markers in parental and integrated genetic maps.

Linkage group	Total markers	Number of markers of segregation distortion	Segregation distortion ratio (%)
LG1	249	0	0
LG2	204	2	0.98
LG3	203	21	10.34
LG4	237	7	2.95
LG5	236	0	0
LG6	239	0	0
LG7	211	12	5.69
LG8	164	0	0
LG9	239	0	0
LG10	144	0	0
LG11	177	0	0
LG12	214	51	23.83
LG13	231	0	0.00
LG14	237	1	0.42
LG15	193	0	0
Total	3,178	0	0

### Visualization and Evaluation of the Genetic Map

**FIGURE 5:**
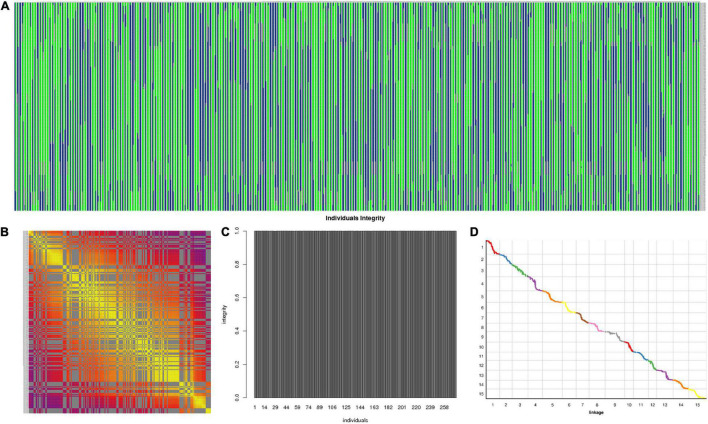
Evaluation of the genetic map. **(A)** Haplotype maps for each individual; **(B)** heatmap of integrated group LG1; **(C)** average integrity of mapping markers; **(D)** collinearity analysis between the linkage map and the corresponding physical map.

The 3,178 SLAF markers were used to construct haplotype maps for each individual, and most of the recombination blocks were identified in the haplotype maps (Figure 5A). Heatmap results indicated a strong linkage relationship between adjacent markers in the linkage group (Figure 5B). The average integrity of mapping markers was 99.98% (Figure 5C). Using the *I. trifida* genome as a reference (Wu et al., 2018), collinearity analysis was performed between the linkage map and the corresponding physical map, the spearman coefficient was close to 1 (Figure 5D and Supplementary Table 5). These results indicated that the genetic map was robust for further QTL mapping.

### Quantitative Trait Loci Associated With Skin Color, Flesh Color, and Anthocyanin Content

We analyzed QTLs for the studied traits in sweet potato based on an integrated genetic map of the 15 LGs of sweet potato spanning 2,233.66 cm. For this purpose, R/qtl software was used, and the regions with LOD scores of 5.6, 5.7, and 5.4 (FC, SC, and AC respectively) were considered as candidate QTL peaks. The LOD threshold was determined with 1,000 permutations.

**FIGURE 6:**
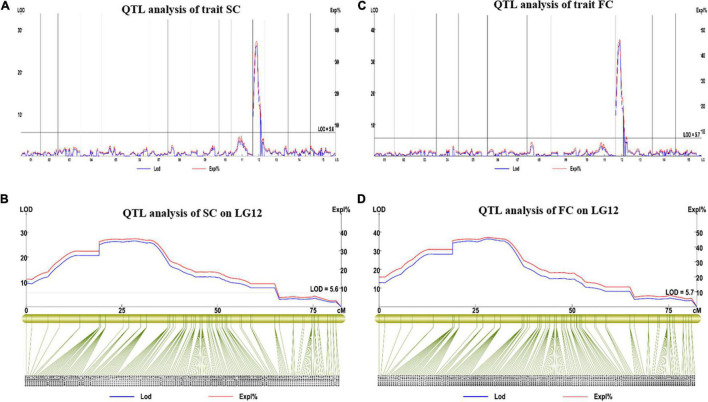
The results of the major QTL test of SC and FC. **(A,B)** Major QTL test results for the SC on LG12; **(C,D)** major QTL test results for the FC on LG12. QTL, quantitative trait loci; SC, skin color; FC, Flesh color.

For SC, we found one major QTL (qSC12-1) in LG12 between 0 and 64.97 cm distance. The LOD value for this trait was observed at 26.29 and explained 36.3% of phenotypic variation (Figures 6A,B). Two SNP markers (3190761 and 3190762) were found in the flanking region of this QTL.

For FC, we found one major QTL (qFC12-1) on LG12 (0–64.97 cM). The LOD value was 35.73, which explained 45.9% of the observed phenotypic variation (Figures 6C,D), its flanking molecular markers were Marker 3190761, Marker 3183569, Marker 3184165, and Marker 3188061. The FC and SC locus was located in the same interval, which was between 4,566,865 and 22,574,309 bp on chromosome 12 of the reference physical map.

**FIGURE 7:**
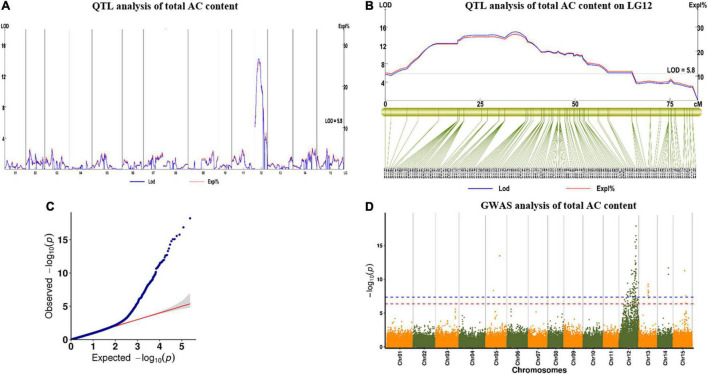
QTL test results of AC by linkage map and GWAS analysis. **(A,B)** QTL analysis for the AC on LG12; **(C)** Quantile-quantile plot. **(D)** Manhattan plot for AC of storage root. The red horizontal dashed line represents the genome-wide significance threshold [–log10(P) = 6.46]. The red triangle corresponds to the proposed functional site. QTL, quantitative trait loci; AC, anthocyanin content; GWAS, genome-wide association study.

For AC in 2019, we found one major QTL (qAC12-1) in YQ and two major QTLs (qAC12-2 and qAC12-3) in ZT on LG12. Their LOD values were 14.22, 18.18, and 6.01, which explained 26, 28.5, and 10.5% of the observed phenotypic variation, respectively (Figure 7 and Table 5). There were 140, 142, and 4 markers closely linked to each QTLs, respectively. For AC in 2020, we found two major QTLs (qAC12-4 and qAC12-5) in YQ and three major QTLs (qAC12-6, qAC12-7, and qAC12-8) in ZT in LG12. Their LOD values were 10.7, 5.74, 4.36, 4.86, 5.18, and 6.01, which explained 22.7, 12.9, 16.7, 18.4, and 19.5% of the observed phenotypic variation, respectively. The peak positions of both qAC12-1 and qAC12-7 are at 25.36 cm. The peak position of both qAC12-2 and qAC12-4 is at 20.76 cm.

**TABLE 5:** Summary of QTLs identified in this study.

Environment	Linkage Group	Name of QTL	QTL Position-start (cM)^a^	QTL Position-end (cM)^b^	Interval	QTL peak Position (cM)*^c^*	Marker number	LOD*^d^*	PVE (%)*^e^*	Flanking molecular markers
2019-YQ	LG12	qAC12-1	5.667	64.968	59.3	25.36	140	14.22	26	Marker3191103, 3190761
2019-ZT	LG 12	qAC12-2	0	64.968	64.97	20.757	142	18.16	28.5	Marker3195593, 3193771, 3195759,3194101, 3193770, 3194100, 3195026,3196512, 3192087
	LG 12	qAC12-3	75.19	75.373	0.18	75.19	4	6.01	10.5	Marker3031695
2020-YQ	LG 12	qAC12-4	8.675	52.477	43.8	20.757	125	10.7	22.7	Marker3193771,3195759, 3194101, 3193770, 3194100, 3195026, 3196512,3192087
	LG 12	qAC12-5	56.244	56.244	0	56.244	1	5.74	12.9	Marker3049324
2020-ZT	LG 12	qAC12-6	43.193	43.193	0	43.193	2	4.36	16.7	Marker3137605,3137036
	LG 12	qAC12-7	19.088	39.014	19.93	25.36	52	4.86	18.4	Marker3191103
	LG 12	qAC12-8	45.445	52.136	6.69	47.28	37	5.18	19.5	Marker3096413,3098398, 3098587

*^a^The start position on the genetic map.*

*^b^The end position on the genetic map.*

*^c^Closely linked or co-localized markers position at the QTL peak.*

*^d^The estimated LOD score at the QTL peak.*

*^e^The proportion of phenotypic variation explained by the QTL.*

*QTL, quantitative trait loci.*

In addition, GWAS was conducted using the results of AC content and SNP marker genotype data. One peak that was highly correlated to AC content was found on LG12 (Figure 7D). This peak was consistent with the major QTL peak on LG12 that was detected *via* QTL analysis (Figures 7A,B), which showed the effectiveness of this QTL.

In conclusion, the major QTLs explaining the observed variation for three color-related traits in the mapping population were co-localized on LG12 between 0 and 64.97 cm, which explained 36.3, 45.9, and 28.6% of the observed variation in SC, FC, and AC, respectively. The co-localized QTLs might be closely linked to each other or the same pleiotropic QTL. The flanking marker (Marker 3190761) was found to be associated with the QTLs for AC, SC, and FC. Phenotyping results indicated that the three traits were highly positively correlated with each other, and highly consistent QTLs explained the phenomenon genetically.

## Discussion

### Development of a High-Quality and High-Density Genetic Map for Sweet Potato Based on SLAF-seq

In this study, a high-quality and high-density SNP linkage map of autohexaploid sweet potato (*I.* batatas L.) was successfully established based on the SLAF-seq technology. In addition, QTLs related to SC, FC, and AC of sweet potato were co-localized on LG12 using this linkage map.

In the past decade, several genetic maps for sweet potatoes have been constructed based on AFLP, RAPD, SSR, and transposon-insertion polymorphism markers (Kriegner et al., 2003; Cervantes-Flores et al., 2008; Zhao et al., 2013; Ma et al., 2020). However, these marker types are poor in repeatability and stability thus limiting the utilization of such kinds of maps.

Next-generation sequencing-based SNP detection accelerated the development of genomic breeding strategies, following the construction of the linkage maps based on SNP markers. Shirasawa et al. established the first high-density genetic map for sweet potato cultivar “Xushu 18” using SNPs identified by double-digest restriction site-associated DNA sequencing using an S1 mapping population. Mollinari et al. built the first multilocus integrated genetic map of an orange-fleshed sweet potato. In this study, we developed SNP markers based on SLAF-Seq and constructed a sweet potato genetic map with 2,233.66 cm in length and an average distance of 0.71 cm. This is the first linkage map of sweet potato-based on color-related traits. Considering the visualization and evaluation of the Genetic Map, the map produced in the present study is higher in density than “xushu18,” which provided a robust prerequisite for QTL identification and mapping.

### Quantitative Trait Loci Analysis of Color-Related Traits in Purple Sweet Potato

The storage root color of the purple sweet potato is determined by FC, SC, and AC. In the past, most studies examining AC accumulation and FC biosynthesis have been performed with a single trait (Kim et al., 2010; Tanaka et al., 2012; Wang et al., 2013; Park et al., 2015). To the best of our knowledge, this is the first research to study the association among the three traits at the genomic level. Fortunately, we performed a QTL analysis and determined one major QTL related to FC, one major QTL related to SC, and eight major QTLs related to AC on LG 12.

Previously, some studies have reported QTLs significantly associated with flesh color in sweet potatoes are located on LG3 and LG12 (Gemenet et al., 2020; Zhang et al., 2020). Zhang et al. (2020) employed GWAS for flesh color in purple sweet potato and determined a QTL on LG12. Similarly, Gemenet et al. (2020) showed the co-localization of major QTLs explaining the observed variation for flesh color and β-carotene on LG3 and LG12. Interestingly, in our study, QTLs for three positively correlated traits were found co-localized on LG12. Particularly, FC and SC, which showed highly consistent results and the physical position of these QTLs, were coincided with the reported, previously (Zhang et al., 2020). This will provide technical guidance for the breeding of sweet potato varieties with high anthocyanin content and ornamental skin color.

Anthocyanin content is a quantitative and complicated trait that is determined by many environmental factors. Although previous studies have shown the IbMYB1-2 is the major gene related to the activation of anthocyanin biosynthesis in the storage roots of flesh color (Kim et al., 2010; Tanaka et al., 2012; Park et al., 2015; Zhang et al., 2020). However, the anthocyanins compositions are complex which include cyanidin, delphinidin 3,5-*O*-diglucoside, malvidin 3,5-*O*-diglucoside, pelargonidin, peonidin, petunidin 3,5-*O*-diglucoside, and other unidentified ingredients (Lee et al., 2013; He et al., 2015). Therefore, extensive genotyping work is required to dissect the genomic regions controlling these different types of anthocyanins.

### Molecular Marker-Assisted Breeding of Purple Sweet Potato

Quantitative trait loci identified in this study were highly consistent and co-localized on the same chromosome. The flanking marker (Marker 3190761) was found to be associated with the QTLs for AC, SC, and FC. Very strong association among FC, SC, AC, and SNP marker 3190761 has strengthened the scope of this marker in marker-assisted (MAS) breeding to aid the selection of sweet potato in early generations. Moreover, a significant association between these QTLs and markers also made it possible to pyramid these QTLs at the same time.

## Data Availability Statement

The original contributions presented in the study are included in the article/Supplementary Material, further inquiries can be directed to the corresponding author/s.

## Author Contributions

HY: conceptualization, software, formal analysis, writing – original draft preparation, and visualization. XW: methodology. HY, MK, and MM: validation. HY, MK, and WS: investigation. WT, HY, and RG: resources. HY and CL: data curation. HY, MAh, and MAr: writing – review and editing. QL: supervision and funding acquisition. YZ and ZL: project administration. All authors have read and agreed to the published version of the manuscript.

## Conflict of Interest

The authors declare that the research was conducted in the absence of any commercial or financial relationships that could be construed as a potential conflict of interest.

## Publisher’s Note

All claims expressed in this article are solely those of the authors and do not necessarily represent those of their affiliated organizations, or those of the publisher, the editors and the reviewers. Any product that may be evaluated in this article, or claim that may be made by its manufacturer, is not guaranteed or endorsed by the publisher.

## Footnotes

1
http://rapdb.dna.affrc.go.jp/
