# Supplementary material for: High-Density Single Nucleotide Polymorphisms Genetic Map Construction and Quantitative Trait Locus Mapping of Color-Related Traits of Purple Sweet Potato [Ipomoea batatas (L.) Lam.]
Source: Front Plant Sci. 2022 Jan 6;12:797041. doi: 10.3389/fpls.2021.797041 (PMC8770336; doi:10.3389/fpls.2021.797041)
Supplement: Supplementary file 1 [file Data_Sheet_1.docx]

Supplementary Table 1 ANOVA analysis of color-related traits in combined environments

| Trait name | Environment | Source | DF | SS | MS | F-value | Pr>F | EstimatedVar |  |
| --- | --- | --- | --- | --- | --- | --- | --- | --- | --- |
| AC | YQ×ZT | Block/Env | 8 | 347.8444 | 43.4805 | 2.9714 | 2.68E-03 | 0.1914 |  |
| AC | YQ×ZT | Genotype | 267 | 1055080.5 | 3951.6123 | 270.044 | 0.00E+00 | 583.2562 |  |
| AC | YQ×ZT | Environment | 3 | 445364.6875 | 148454.8906 | 10145.0635 | 0.00E+00 | 328.2261 |  |
| AC | YQ×ZT | GE_interaction | 332 | 413470.375 | 1245.3927 | 85.1073 | 0.00E+00 | 729.339 |  |
| AC | YQ×ZT | LSD(0.01) | 76.042 |  |  |  |  |  |  |
| AC | YQ×ZT | H^2_per_plot | 0.4395 |  |  |  |  |  |  |
| AC | YQ×ZT | H^2_per_mean | 0.7597 |  |  |  |  |  |  |
| SC | YQ×ZT | Genotype | 269 | 710.6189 | 2.6417 | Infinity | 0.00E+00 | 0.2201 |  |
| SC | YQ×ZT | Environment | 3 | 0 | 0 | / | 1.00E+00 | 0 |  |
| SC | YQ×ZT | GE_interaction | 807 | 0 | 0 | / | 1.00E+00 | 0 |  |
| SC | YQ×ZT | LSD(0.01) | 0 |  |  |  |  |  |  |
| SC | YQ×ZT | H^2_per_plot | 1 |  |  |  |  |  |  |
| SC | YQ×ZT | H^2_per_mean | 1 |  |  |  |  |  |  |
| FC | YQ×ZT | Genotype | 269 | 2511.0732 | 9.3348 | Infinity | 0.00E+00 | 0.7779 |  |
| FC | YQ×ZT | Environment | 3 | 0 | 0 | / | 1.00E+00 | 0 |  |
| FC | YQ×ZT | GE_interaction | 807 | 0 | 0 | / | 1.00E+00 | 0 |  |
| FC | YQ×ZT | LSD(0.01) | 0 |  |  |  |  |  |  |
| FC | YQ×ZT | H^2_per_plot | 1 |  |  |  |  |  |  |
| FC | YQ×ZT | H^2_per_mean | 1 |  |  |  |  |  |  |
|  |  |  |  |  |  |  |  |  |  |

Note: DF: The degree of freedom of variance; SS: Sum of Squares; MS: The mean square of variance; F: Value of F-test; P: P-value of F-test.

Supplementary Table 2 Marker Number in the linkage group

| Linkage group | Marker Number | Linkage group | Marker Number |
| --- | --- | --- | --- |
| LG1 | 299 | LG9 | 296 |
| LG2 | 261 | LG10 | 209 |
| LG3 | 252 | LG11 | 199 |
| LG4 | 369 | LG12 | 276 |
| LG5 | 259 | LG13 | 268 |
| LG6 | 284 | LG14 | 259 |
| LG7 | 250 | LG15 | 231 |
| LG8 | 211 | Total | 3,923 |

Supplementary Table 3 Summary of the 15 linkage groups of ‘Xuzishu8’

| LG ID | MarkerNum | Total Distance | Average Distance | Gaps<=5 | Max Gap |
| --- | --- | --- | --- | --- | --- |
| Chr01 | 162 | 119.50 | 0.74 | 97.52% | 11.86 |
| Chr02 | 121 | 117.76 | 0.98 | 96.67% | 16.24 |
| Chr03 | 121 | 130.35 | 1.09 | 94.17% | 17.78 |
| Chr04 | 43 | 111.81 | 2.66 | 90.48% | 34.29 |
| Chr05 | 119 | 200.82 | 1.70 | 94.92% | 30.80 |
| Chr06 | 121 | 141.51 | 1.18 | 95% | 25.06 |
| Chr07 | 104 | 102.26 | 0.99 | 99.03% | 14.75 |
| Chr08 | 63 | 153.72 | 2.48 | 95.16% | 54.57 |
| Chr09 | 108 | 221.59 | 2.07 | 94.39% | 114.05 |
| Chr10 | 66 | 62.31 | 0.96 | 95.38% | 21.57 |
| Chr11 | 104 | 150.63 | 1.46 | 93.2% | 19.37 |
| Chr12 | 139 | 79.27 | 0.57 | 97.1% | 16.75 |
| Chr13 | 123 | 147.25 | 1.21 | 94.26% | 18.30 |
| Chr14 | 93 | 131.90 | 1.43 | 92.39% | 17.26 |
| Chr15 | 99 | 180.65 | 1.84 | 92.86% | 64.12 |
| Total | 1,586 | 2,051.33 | 1.31 | 94.84% | 114.05 |

Supplementary Table 4 Summary of the 15 linkage groups of ‘Meiguohong’

| LG ID | MarkerNum | Total Distance | Average Distance | Gaps<=5 | Max Gap |
| --- | --- | --- | --- | --- | --- |
| Chr01 | 96 | 127.03 | 1.34 | 94.74% | 26.28 |
| Chr02 | 93 | 123.54 | 1.34 | 93.48% | 20.46 |
| Chr03 | 88 | 169.94 | 1.95 | 91.95% | 36.52 |
| Chr04 | 202 | 163.51 | 0.81 | 98.51% | 7.46 |
| Chr05 | 123 | 133.05 | 1.09 | 95.9% | 15.74 |
| Chr06 | 121 | 110.88 | 0.92 | 96.67% | 32.17 |
| Chr07 | 113 | 144.11 | 1.29 | 97.32% | 29.46 |
| Chr08 | 107 | 138.03 | 1.30 | 96.23% | 34.29 |
| Chr09 | 143 | 140.17 | 0.99 | 95.77% | 21.57 |
| Chr10 | 85 | 97.14 | 1.16 | 92.86% | 10.98 |
| Chr11 | 79 | 130.79 | 1.68 | 89.74% | 23.61 |
| Chr12 | 86 | 63.95 | 0.75 | 97.65% | 18.84 |
| Chr13 | 115 | 164.26 | 1.44 | 96.49% | 34.29 |
| Chr14 | 148 | 162.87 | 1.11 | 96.6% | 25.06 |
| Chr15 | 101 | 154.91 | 1.55 | 93% | 22.13 |
| Total | 1,700 | 2,024.18 | 1.20 | 95.13% | 36.52 |

Supplementary Table 5 Spearman coefficient between linkage group and genetic map

| Linkage group | Spearman | Linkage group | Spearman |
| --- | --- | --- | --- |
| LG1 | 0.9971 | LG9 | 0.9926 |
| LG2 | 0.9975 | LG10 | 0.9925 |
| LG3 | 0.9643 | LG11 | 0.9990 |
| LG4 | 0.9918 | LG12 | 0.9918 |
| LG5 | 0.9952 | LG13 | 0.9843 |
| LG6 | 0.9950 | LG14 | 0.9962 |
| LG7 | 0.9968 | LG15 | 0.9959 |
